# Drug-Induced Immune Hemolytic Anemia Following Dapagliflozin Administration: A Case Report

**DOI:** 10.7759/cureus.79630

**Published:** 2025-02-25

**Authors:** Bergen Lemack, Gabriel Kupovics, Rohit Kumar

**Affiliations:** 1 College of Medicine, Texas College of Osteopathic Medicine, Fort Worth, USA; 2 Internal Medicine, Texas Health Huguley Hospital, Burleson, USA

**Keywords:** adverse drug reaction, dapagliflozin, drug-induced immune hemolytic anemia, hemolytic anemia, jaundice

## Abstract

Drug-induced immune hemolytic anemia (DIIHA) is an extremely rare and often undiagnosed cause of anemia. Due to variability in antibody type, binding affinity, and the presence or absence of the drug at the time of testing, serologic findings can be inconsistent, making diagnosis challenging and delaying treatment, which increases the risk of progression to organ failure or death. In this case report, we discuss a 52-year-old Caucasian male being treated for type 2 diabetes mellitus (T2DM) with dapagliflozin who developed jaundice and progressive fatigue after two weeks of treatment. He was found to have evidence of extravascular hemolysis with transaminitis, indirect hyperbilirubinemia, elevated LDH, and decreased haptoglobin. The patient was diagnosed with DIIHA likely due to dapagliflozin, and the medication was immediately discontinued from his regimen in the emergency department. He was stabilized after two transfusions of packed red blood cells (RBCs) and a short course of glucocorticoids. Here, we discuss the pathophysiology, workup, and management of DIIHA, a previously unreported adverse reaction to dapagliflozin, a drug commonly used to treat T2DM.

## Introduction

Drug-induced immune hemolytic anemia (DIIHA) is a rare cause of anemia with an incidence of one or two per million per year worldwide [1-3]. When unrecognized and unaddressed, it can cause severe hemolysis leading to life-threatening shock, organ ischemia, disseminated intravascular coagulation (DIC), and acute respiratory distress syndrome (ARDS) [3]. DIIHA is most commonly associated with antibiotics, particularly those in the penicillin and cephalosporin classes, but cases have been identified in more than 138 medications in total [1,4].

Dapagliflozin (Farxiga) is a sodium-glucose cotransporter 2 (SGLT2) inhibitor commonly used in the treatment of type 2 diabetes mellitus (T2DM). While generally well-tolerated, it is associated with increased glucose excretion in the urine, which can predispose patients to various urinary tract infections [5]. However, dapagliflozin-induced hemolytic anemia is not yet reported in the literature. In this case, we introduce a 52-year-old patient who developed hemolytic anemia two weeks after initiating dapagliflozin for the treatment of T2DM. We then discuss the pathophysiology and a therapeutic solution for DIIHA to bring awareness to the potentially unrecognized adverse drug effect.

## Case presentation

A 52-year-old Caucasian male with a history of T2DM, hyperlipidemia, chronic kidney disease (CKD, stage 1), hypothyroidism, and previous kidney stones was brought to the emergency department (ED) by his wife due to noticeable jaundice and fatigue. The patient reported a one-week history of progressive fatigue, exertional dyspnea, headaches, and dark urine with dull, constant right flank pain without dysuria or hematuria. He noted this pain was different from his prior kidney stone episodes. He denied nausea, vomiting, diarrhea, weight loss, fever, or chills. He denied recent travel outside the U.S. or unusual food consumption.

Two weeks prior to the onset of symptoms, he began taking dapagliflozin 5 mg, prescribed by the nephrology team managing his CKD and T2DM on an outpatient basis. The patient expressed a poor history of tolerating new medications due to unwanted side effects, specifically mentioning failed treatment with statin therapy for hyperlipidemia. His home medications included insulin (Lantus and aspart), metoprolol, fenofibrate (due to statin intolerance), vitamin D3, and B12 supplements. His social history revealed chewing tobacco use and one alcoholic beverage per week. In the ED, dapagliflozin was discontinued due to concerns about its potential role in his symptoms.

Initial workup (day 1)

Initial labs in the ED showed increased total bilirubin with elevated indirect bilirubin, elevated glucose, mildly elevated AST and ALT, and decreased hemoglobin and hematocrit. Lipid profile revealed hypertriglyceridemia and decreased HDL cholesterol. Iron studies showed increased iron levels, ferritin, and iron saturation, with normal total iron-binding capacity and transferrin. The patient’s vitamin B12 level was markedly elevated. Renal function was unremarkable except for a mildly elevated blood urea nitrogen (BUN) of 24.3. Protein electrophoresis and folate levels were unremarkable. Importantly, CT imaging of the abdomen and pelvis showed no splenomegaly or signs of lymphoproliferative disorder. An EKG showed sinus tachycardia but was otherwise normal, with no acute changes from baseline.

Day 2

On day 2, labs showed worsening indirect bilirubin. Lactate dehydrogenase (LDH) was measured at this time and was elevated. The patient's symptoms worsened, with complaints of exertional dizziness, lightheadedness, and headaches. He remained afebrile, but his hemoglobin decreased further. A peripheral blood smear showed slight anisocytosis, no schistocytes, moderate hypochromia, slight polychromasia, and rare large platelets. Coombs' test was ordered, which was negative. On day 3, due to ongoing anemia and worsening symptoms, the patient was given a single unit of packed red blood cells (PRBC). His hemoglobin increased slightly following transfusion, and the corrected reticulocyte count was measured at 0.71%. A corrected reticulocyte count is calculated to account for the degree of anemia, providing a more accurate representation of bone marrow response

In addition, the patient's thyroid function tests revealed elevated thyroid-stimulating hormone (TSH), consistent with his history of hypothyroidism. The gastrointestinal (GI) team ruled out any GI causes for his jaundice based on lab findings and imaging. Hepatitis A, B, and C panels were negative, ruling out viral hepatitis as a cause of his jaundice and liver dysfunction. Antinuclear antibody (ANA) testing was positive, suggesting a possible autoimmune component contributing to his presentation.

Progression and treatment (days 3-5)

By day 3, the patient’s hemoglobin had decreased further. He experienced worsening symptomatic jaundice, with new complaints of lightheadedness when walking to the bathroom, leading to nausea and vomiting in the shower. His symptoms were managed with rest. His BUN remained elevated, and his glucose levels ranged from elevated to very high. His LDH increased further. C3 and C4 complement levels were measured and were normal. The patient was still reporting dark red urine color.

On day 4, worsening hemolysis was noted, as his hemoglobin remained low, and he continued to experience ongoing fatigue, new-onset nausea, vomiting, and worsening jaundice. Prednisone 50 mg twice daily was initiated on day 4 due to concerns of an autoimmune process contributing to hemolysis. In addition, a second PRBC transfusion was given. The consulting hematologist decided to initiate prednisone as a precautionary measure due to the rapid decline in hemoglobin and worsening clinical symptoms.

By day 5, his reticulocyte count had increased, indicating a more appropriate erythropoietic response. A third PRBC transfusion was administered on day 5 for symptomatic anemia, and the patient’s hemoglobin improved slightly. During this time, his glucose remained elevated, and his bilirubin continued to rise, with total bilirubin reaching its highest level.

Recovery (days 6-7)

By day 6, the patient reported feeling the best he had since admission, with improvement in his jaundice and resolution of his lightheadedness, nausea, and vomiting. His urine was now described as “dehydrated yellow” without an orange tint. Despite ongoing prednisone treatment, his hemoglobin remained low, with further increases in reticulocyte count indicating a stronger erythropoietic response. His BUN continued to trend upwards, and glucose levels remained erratic and high.

At discharge on day 7, BUN and glucose were still elevated, and hemoglobin had slightly decreased. Total bilirubin had stabilized with a persistent indirect component. The patient was discharged on prednisone 50 mg twice daily with close hematology follow-up. Detailed laboratory findings are presented in Table 1.

**Table 1:** Outline of the patient’s laboratory results BUN = blood urea nitrogen, AST = aspartate aminotransferase, ALT = alanine transaminase, DBIL = direct bilirubin, IBIL = indirect bilirubin, LDH = lactate dehydrogenase, RBC = red blood cells, Hgb = hemoglobin

Lab parameter (units)	Day 1 PM (arrival)	Day 2 AM (first transfusion)	Day 2 PM	Day 3 AM	Day 3 PM	Day 4 AM (prednisone + second transfusion)	Day 5 AM	Day 5 PM (third transfusion)	Day 6 AM	Day 6 PM	Day 7 AM (discharge)	Reference range
Glucose (mg/dL)	262	210	-	225	-	244	297	-	282	-	310	70–110
BUN (mg/dL)	24.3	21.4	-	21.7	-	24	29.8	-	34.9	-	35.5	7–20
Creatinine (mg/dL)	1.14	0.81	-	0.72	-	0.9	1.0	-	1.12	-	1.15	0.6–1.2
AST (U/L)	47	35	-	43	-	45	-	-	-	-	-	5–40
ALT (U/L)	64	52	-	52	-	54	-	-	-	-	-	7–56
DBIL (mg/dL)	1.09	0.9	-	1.2	-	1.6	-	-	-	-	-	0.0–0.3
IBIL (mg/dL)	3.61	3.8	-	4.5	-	4.3	-	-	-	-	-	0.1–1.0
LDH (U/L)	-	354	-	454	-	-	-	-	-	-	-	140–280
RBC (million/μL)	2.46	2.03	-	2.09	2.02	1.84	2.09	-	2.45	-	2.25	4.2–5.9
Hgb (g/dL)	7.7	6.2	7.3	6.2	6.2	6.6	6.6	7.7	7.4	7.2	7.1	13.5–17.5
Hematocrit (%)	21.8	18.0	21.3	18.4	17.6	18.7	18.0	21.8	22.3	21.2	21.2	40–50
Reticulocyte count (%)	-	-	1.37	1.51	-	1.64	2.65	-	4.44	-	-	0.5–2.5
Corrected reticulocyte Count (%)	-	-	0.71	0.62		0.68	1.06	-	2.2	-	-	0.5–2.5

## Discussion

DIIHA is a rare cause of anemia that develops hours to months after exposure to a pharmaceutical agent. The reaction can result after the first exposure or repeated use of medication, with the latter scenario risking a more rapid, life-threatening hemolysis. More than 138 drugs have been associated with DIIHA, and the number of recognized cases continues to grow as new medications enter the market [1]. The drug classes most frequently associated with DIIHA are antimicrobials, particularly cephalosporins and penicillins, non-steroidal anti-inflammatory drugs, and platinum-based chemotherapeutics [2]. Because laboratory results in patients with DIIHA can present similarly to those expected in autoimmune hemolytic anemia (AIHA)or hemolytic transfusion reactions, cases of DIIHA may go undetected [6]. For this reason, it is important to be aware of the pathophysiology and associated drugs for early recognition and treatment initiation.

The pathophysiology of DIIHA is primarily the result of antibody production in response to drug exposure. Antibody development can be further characterized as drug-independent (DIABs) or drug-dependent (DDABs) [2,7]. DIABs may complex with RBCs and induce hemolysis in the absence of an inciting agent, whereas DDABs require the presence of the drug in order to bind and lyse RBCs [2,8]. The mechanism(s) of hemolysis that constitute each case results in varied serology results, which can complicate diagnosis.

Drug-dependent DIIHA tends to present within minutes or hours of drug exposure and can manifest as either intra- or extravascular hemolysis, which is contingent upon whether the complement system is activated [3,8-10]. In addition to the acute onset, these cases tend to present as more profound hemolysis with higher rates of decompensated heart failure, acute renal failure and shock, DIC, and death [1-3,9], none of which were seen in this patient.

A milder, sub-acute presentation is typically seen in drug-independent DIIHA and is characterized by signs and symptoms of anemia after days to weeks of drug exposure and can occur in the absence of the offender itself. In this case, moderate symptoms emerged after two weeks of initiating dapagliflozin and persisted for a few days following discontinuation. This clinical course aligns with the mechanism of drug-independent DIIHA described in the literature. While in the bloodstream, the drug loosely interacts with the RBC membrane modifying its normal structural components and altering RBC surface antigens [11,12]. DIABs may then interact and bind the RBC membrane in the absence of the inciting agent and induce extravascular hemolysis in the spleen and reticuloendothelial system independent of complement activation [9]. These autoantibodies are serologically identical to those seen in warm AIHA (WAIHA), so differentiation between the two conditions is dependent upon improved patient condition with discontinuation of the suspected offending agent [2,3,7,13-15].

Symptoms of DIIHA are commonly vague initially, but with progression, they become indicative of an underlying hemolytic anemia. Patients may experience fatigue, weakness, dizziness, palpitations, dyspnea, and/or hemoglobinuria. Jaundice, pallor, hepatomegaly, splenomegaly, and adenopathy may also be seen on physical exam [15,16]. As mentioned previously, more serious complications may emerge, which necessitates the collection of a thorough medical and drug history for early identification of potential causative agents [16,17]. The patient in this case presented with complaints of progressive fatigue, exertional dyspnea, dark urine, and worsening jaundice, all of which prompted the investigation of hemolytic anemia as the cause.

Diagnosis of DIIHA involves a history of intake of an offending medication and clinical and laboratory evidence of hemolysis [10,15]. Similar to other hemolytic anemias, laboratory analysis will be characterized by significant reductions in hemoglobin and hematocrit, with normocytic mean corpuscular volumes (MCV) (80-100 fL). Reticulocyte count may be reduced acutely followed by a reactive elevation from increased RBC production [3]. Hemolysis may also result in elevated LDH, ferritin, LFTs, and indirect bilirubin with significantly decreased haptoglobin [10,18]. Peripheral blood smear (PBS) may help identify a mechanism of hemolysis. For example, PBS that reveals spherocytes suggests immune hemolysis, whereas schistocytes indicate thrombotic microangiopathy and bite cells, blister cells, and/or poikilocytosis favor a mechanism of oxidative hemolysis [11,18]. Differentiation between immune and non-immune-mediated etiologies of hemolysis is investigated with a direct Coombs’ test (DAT). A positive result suggests an immune-mediated hemolytic reaction and is more likely in DIIHA when the causative drug is actively being administered. Conversely, the test can be negative if no drug is present, but the resulting hemolysis-inducing antibody is already bound to RBC membranes [19]. As a result, many cases of identified DIIHA, including this one, have reported negative DAT results [12,17].

The diagnosis of DIIHA for the patient in this case was further supported by the patient's clinical presentation and laboratory findings, including decreased haptoglobin, elevated LDH, increased reticulocyte count, and indirect hyperbilirubinemia, in conjunction with the temporal relationship to dapagliflozin initiation. PBS revealed anisocytosis and polychromasia while his reticulocyte count increased throughout admission, indicating appropriate bone marrow response to hemolysis and associated spherocytosis, which further supports a hemolytic anemia diagnosis. While other etiologies such as hemoglobinopathies and lymphoproliferative disorders were ruled out based on normal CT imaging and lab findings, a bone marrow biopsy was not performed due to the lack of specific indications for this patient.

DAT was negative while antinuclear antibody (ANA) resulted positive indicating the presence of autoantibodies with weak affinity for RBC antigens. The half-life of dapagliflozin is 12.9 hours; meaning, the drug was likely cleared before Coombs’ testing, which could have contributed to a negative DAT [20]. While the presence of an autoantibody necessitates consideration of WAIHA, as previously stated, it does not exclude DIIHA due to DIABs. Normal complement levels seen in this case point to a drug-independent mechanism of DIIHA in which DIABs that formed during drug administration continue to exist in vivo after discontinuation of the agent. These DIABs could have then complexed with RBC membranes to induce extravascular hemolysis independent of complement activation. This cause of hemolytic anemia is further supported by the lack of signs of hemolytic anemia or autoimmune phenomena prior to the administration of dapagliflozin. The patient’s medical history, combined with continued clinical improvement with cessation of the suspected culprit, supports the diagnosis of DIIHA secondary to dapagliflozin.

In the management of DIIHA, cessation of the incriminating drug is the most effective intervention and is always recommended [1,11,16]. Blood transfusion is also indicated in cases where hemoglobin levels fall below 7.0 g/dL. In patients presenting with drug-independent DIIHA, a one- to three-week course of glucocorticoids may be added, either orally or intravenously (for cases of severe, life-threatening hemolysis) [1,7]. Steroid treatment of DIIHA characterized by DIABs (e.g., dapagliflozin and fludarabine) has shown a 75-96% response rate, but there is little data concerning the efficacy of steroids in cases caused by DDABs (e.g., penicillins and cephalosporins) [1]. Severe and relapsing cases can be further managed with IVIG therapeutic plasma exchange and/or immunomodulatory therapy, such as rituximab or cyclophosphamide [1,11,13,15]. In most cases, clinical improvement is seen within one to two weeks after discontinuation of the drug and initiation of the aforementioned therapy options. The initiation of glucocorticoids was a judgment call aimed at mitigating worsening anemia, with plans for ongoing outpatient hematology evaluation to confirm the etiology and adjust treatment as necessary. Although the patient's anemia and laboratory values were not fully resolved by discharge, several indicators of improvement were observed, including reduced jaundice, resolution of symptoms such as nausea and lightheadedness, and a steadily increasing reticulocyte count, reflecting an appropriate bone marrow response. This case highlights the importance of clinical judgment in the management of suspected DIIHA. Future avoidance of the medication and others in the same pharmaceutical class is highly recommended as cross-reactivity can occur [3,8]. Patients should also be encouraged to follow up with hematology in an outpatient setting for continued monitoring of their condition.

## Conclusions

This case emphasizes the critical importance of recognizing DIIHA as a rare but potentially life-threatening cause of anemia. Although infrequent, DIIHA requires prompt diagnosis and management due to severe complications. Clinical and laboratory findings often mimic other forms of hemolytic anemia, such as AIHA, making diagnosis challenging. A thorough drug history is essential for identifying potential offending agents, especially as the list of medications associated with DIIHA continues to expand. Early discontinuation of the suspected drug and supportive care, including corticosteroids and transfusions as needed, are necessary for treatment. This case emphasizes the importance of maintaining a high index of suspicion for DIIHA in patients presenting with hemolysis and recent medication changes to improve outcomes and avoid delays in care.
